# Association between infant crawling characteristics and fundamental movement skill development in preschool children

**DOI:** 10.3389/fpsyg.2026.1944438

**Published:** 2026-10-15

**Authors:** Yuehui Zhou, Huizi Yin, Yingxiang Fang, Bingao Chen, Juhua Wu, Yang Yuan

**Affiliations:** 1 School of Sport Science, Qufu Normal University, Qufu, Shandong, China; 2 School of Physical Education, Northeast Normal University, Jilin, China; 3 Yantai Development Zone Experimental Middle School, Shandong, China; 4 School of Sports,Yunnan Normal University, Kunming, China; 5 School of Sports, Guangxi University of Science and Technology, Liuzhou, Guangxi, China

**Keywords:** crawling experience, fundamental movement skills, infant crawling, insufficient crawling experience, preschool children

## Abstract

This study examined the associations between infant crawling experience and crawling characteristics and fundamental movement skill (FMS) development in preschool children to characterize population-level associations and generate hypotheses for prospective research. A retrospective cross-sectional design was used, including 557 children aged 3–6 years from kindergartens in Qufu, Shandong Province, and the surrounding areas. Infant crawling experience and crawling characteristics were collected retrospectively through guardian questionnaires, and FMS was assessed using the Test of Gross Motor Development–Third Edition (TGMD-3). Children with a total FMS score below the 25th percentile were classified as having relatively insufficient FMS development, and a multivariable logistic regression analysis was performed to examine these associations. Compared with children with relatively sufficient crawling experience, those with insufficient crawling experience had lower locomotor skill and ball skill scores, as well as lower total FMS scores. These findings suggest that insufficient crawling experience and selected crawling characteristics, particularly daily crawling duration and crawling-posture diversity, are statistically associated with relatively insufficient FMS development in preschool children; however, their significance requires further confirmation in prospective studies.

## 1 Introduction

Fundamental movement skills (FMS) are basic motor abilities that develop progressively during childhood and commonly include locomotor skills, object control skills, and balance or stability skills (Rudd et al., 2015; Stodden et al., 2008). These skills provide an important foundation for children’s participation in play, sports, and habitual physical activity and support later participation in more complex motor activities and sustained physically active behavior. Previous studies have shown that FMS proficiency is positively associated with physical activity, cardiorespiratory fitness, and health-related fitness and is negatively associated with unfavorable weight status (Utesch et al., 2019; Lubans et al., 2010). Developmental models of motor competence further suggest that there are dynamic reciprocal relationships among motor competence, physical activity, health-related fitness, and weight status (Schmutz et al., 2020). Additionally, lower motor competence early in life may be associated with reduced physical-activity participation, as well as subsequent fitness and health outcomes (Stodden et al., 2008). Identifying early factors associated with FMS development is therefore important for motor development assessment, early intervention, and health promotion. The preschool period is a critical stage for rapid FMS development and for the continued accumulation of coordination, postural control, and movement experience. Existing research has primarily examined factors during the preschool period, including age, sex, body mass index (BMI), physical activity, family environment, kindergarten physical activity, and motor interventions (Barnett et al., 2013; Capelle et al., 2017; Engel et al., 2018; Zhang et al., 2022). However, children have already undergone a substantial period of motor development before entering kindergarten, and infant postural control, self-produced locomotion, and environmental exploration may be relevant to the later development of more complex skills such as running, jumping, throwing, and catching. A focus limited to preschool-age factors may therefore be insufficient to explain variations in FMS development, supporting the need to extend the research perspective to infancy.

Crawling is an important form of early self-produced locomotion and is one of the motor milestones that may occur during gross motor development. The World Health Organization Multicentre Growth Reference Study identified hands-and-knees crawling as one of the six gross motor milestones, highlighting crawling as a component of early motor development and independent mobility experience (World Health Organization Multicentre Growth Reference Study Group, 2006). As a form of self-produced locomotion, crawling provides infants with opportunities for independent movement and environmental exploration. Such locomotor experience may be relevant to later motor development, although the specific mechanisms remain unknown. Kretch et al. (2014) have shown that crawling and walking infants differ in their visual experiences during natural locomotion, suggesting that self-produced locomotor mode may influence how infants explore the environment and obtain perceptual information. Franchak and Adolph (2024) further emphasized substantial individual variability in the timing and developmental trajectories of infant motor skills; crawling and walking milestones therefore should not be interpreted as a rigid sequence that all children acquire at fixed ages. Population-based evidence has also suggested that a mildly delayed onset of crawling and independent walking is associated with later motor impairments (Hua et al., 2022), indicating a possible association between early motor milestones, self-produced locomotor experience, and later motor development. Importantly, “whether a child crawled” and “insufficient infant crawling experience” are not equivalent concepts. “Whether a child crawled” indicates only whether crawling occurred before independent walking, whereas “crawling experience” also encompasses duration, daily exposure, and movement forms. Accordingly, the present study uses “insufficient crawling experience” and “relatively sufficient crawling experience” to describe the two principal groups; the operational criteria are specified in the Methods section. Chaplais and Macfarlane (1984) used a total crawling duration of less than 2 months (<60 days) to characterize absent or significantly limited crawling experience among late-walking children; however, definitions vary across studies, and this historical threshold should not be interpreted as a universally validated clinical definition. Previous studies have suggested that absent or limited crawling experience may be associated with later motor performance (Visser and Franzsen, 2010). In the present study, the term “insufficient crawling experience” therefore refers to the relative sufficiency of self-produced crawling experience during infancy.

Because infant crawling precedes the current assessment of FMS, relevant information in studies of preschool children often must be collected retrospectively from parents or primary caregivers. Parent reports of gross motor milestone attainment have an established role in developmental research, and previous evidence has suggested that reports of salient gross motor milestones can provide useful information (Bodnarchuk and Eaton, 2004). Parent-report tools, such as the Early Motor Questionnaire (EMQ) and the Ages and Stages Questionnaires, Third Edition (ASQ-3), have also been used to assess early motor development (Libertus and Landa, 2013; Squires and Bricker, 2009). Nevertheless, evidence specifically linking insufficient infant crawling experience with FMS in preschool children remains limited. Previous studies have often treated infant motor milestones as general developmental indicators and have rarely examined insufficient crawling experience as a distinct early locomotor experience variable. Even studies focusing on crawling have often used a simple crawled/did-not-crawl comparison, with less attention to the sufficiency of crawling experience or to characteristics such as daily crawling duration, the interval from crawling onset to independent walking, crawling posture, and crawling motivation in relation to later FMS (Cazorla-González et al., 2022). Accordingly, the primary hypothesis of this retrospective cross-sectional study was that children with insufficient crawling experience would have higher odds of being classified as having relatively insufficient FMS development than those with relatively sufficient crawling experience. We additionally examined associations of daily crawling duration, the interval from crawling onset to independent walking, and crawling posture with relatively insufficient FMS development, whereas crawling motivation, backward crawling, and crawling while carrying objects were treated as exploratory analyses.

## 2 Materials and methods

### 2.1 Participants

Children aged 3–6 years were included in this study primarily from junior, middle, and senior classes at kindergartens in Qufu, Shandong Province, and the surrounding areas. The inclusion criteria were as follows: (1) age 3–6 years; (2) generally healthy physical and psychological status, with no intellectual disability, motor impairment, congenital disease, or chronic disease; (3) no contraindication to physical activity; and (4) informed consent provided by a guardian. Data were collected from early April to late June 2024. After screening, 557 children were included in the statistical analyses, including 295 boys and 262 girls. The study was approved by the Bioethics Committee of Qufu Normal University (approval no. 2024.0253), and written informed consent was obtained from the guardians of all participating children.

### 2.2 Study design

A retrospective cross-sectional design was used. Infant crawling history and specific crawling characteristics were collected retrospectively using guardian questionnaires, and current FMS was assessed using the Test of Gross Motor Development–Third Edition (TGMD-3). Infant crawling-experience status was the primary exposure, and relatively insufficient FMS development was the primary outcome. A multivariable logistic regression analysis was performed to examine their associations, with supplementary analyses of daily crawling duration, the interval from crawling onset to independent walking, crawling posture, crawling motivation, backward crawling, and crawling while carrying objects. Sensitivity analyses were performed to evaluate the robustness of the findings. Receiver operating characteristic (ROC) curves were generated as an exploratory analysis to describe the apparent discrimination of the fitted logistic regression models within the present sample. This analysis was not intended to develop or validate a clinical prediction model, and no internal or external model validation was performed. Although infant crawling occurred before the current FMS assessment, the exposure information was reported retrospectively by guardians, and the data were obtained from a one-time questionnaire and on-site assessment. Accordingly, this study evaluated statistical associations only and did not support causal inference or individual-level developmental-trajectory inference.

### 2.3 Measures and definitions

#### 2.3.1 Infant crawling experience and crawling characteristics

Infant crawling history and crawling characteristics were reported retrospectively by guardians using a questionnaire developed for the original study. Based on prespecified questionnaire items, we constructed an operational composite indicator of “insufficient crawling experience” comprising the following: (1) a total crawling duration of less than 2 months (<60 days), adapted from the threshold used by Chaplais and Macfarlane (1984); (2) no crawling before transition directly to standing or walking; (3) being carried for much of infancy with relatively few opportunities for self-produced crawling; (4) guardian-reported crawling movements that appeared awkward or effortful; and (5) use of a baby walker or other childcare equipment that restricted independent floor activity for >2 h/day. Children meeting any one of these items were classified as having insufficient crawling experience; all others were classified as having relatively sufficient crawling experience. This classification reflects only the relative sufficiency of self-produced crawling experience and is an operational research definition rather than a clinical diagnostic criterion; the composite indicator has not undergone independent psychometric validation. All information was based on guardians’ retrospective reports of infant behavior when the participating children were 3–6 years old. To facilitate recall, guardians were encouraged to consult available family records, such as child-growth record applications, infant photographs or home videos, and child health records when completing the questionnaire. During questionnaire quality control, logically related items, including whether the child had crawled and the reported duration of crawling, were cross-checked for internal consistency; discrepant responses were verified when possible or excluded when inconsistencies could not be resolved. Previous research has suggested that parent reports of salient gross motor milestones can provide useful information (Bodnarchuk and Eaton, 2004). However, the evidence from Bodnarchuk and Eaton (2004) was based on structured, relatively proximal recording of motor milestones and is not fully equivalent to the multi-year retrospective recall used in the present study. Daily crawling duration and the interval from crawling onset to independent walking were recorded using prespecified categories rather than requiring exact recalled times; nevertheless, retrospective recall bias and exposure misclassification cannot be excluded.

#### 2.3.2 Assessment of fundamental movement skills and definition of relatively insufficient development

Children’s FMS were assessed using the TGMD-3. The TGMD-3 comprises two domains—locomotor skills and ball skills—and 13 motor skills in total, including six locomotor skills and seven ball skills. Each skill is scored against specific performance criteria, with 1 point awarded when a criterion is demonstrated and 0 when it is not; each skill is assessed across two scored trials. The maximum raw scores are 46 for locomotor skills, 54 for ball skills, and 100 for the total FMS score (Ulrich, 2019; Webster and Ulrich, 2017; Rey et al., 2020). Studies in Chinese children have reported good applicability, internal consistency, test–retest reliability, and construct validity of the TGMD-3, supporting its use to assess FMS in Chinese children (Diao et al., 2018; Li et al., 2022). Assessments were administered by uniformly trained examiners using standardized demonstrations, instructions, and scoring criteria. The sample-specific 25th percentile (P25) was used to characterize relative FMS performance. In the present sample, P25 corresponded to a total FMS score of 47; children with scores <47 were classified as having “relatively insufficient FMS development,” whereas those with scores ≥47 were classified as “not relatively insufficient.” This cutoff was used only to identify relatively low FMS scores within the present sample and represents a sample-specific statistical classification rather than an official TGMD-3 normative cutoff or a clinical diagnostic threshold.

#### 2.3.3 Covariates

Covariates were selected based on previous research and data availability and included age, sex, BMI, gestational age, feeding mode, mode of delivery, highest parental educational level, paternal smoking history, and pregnancy complications. Age was recorded in years at the time of testing, and sex was classified as either boy or girl. BMI was calculated as weight/height^2^ (kg/m^2^). Gestational age was categorized as <37 weeks, 37–41 weeks, or ≥42 weeks. Feeding mode was categorized as breastfeeding, formula feeding, or mixed feeding. Mode of delivery was categorized as vaginal or cesarean delivery. The highest parental educational level was defined as the higher educational attainment of the two parents and categorized as junior high school or below, high school or technical secondary school, college/university, or master’s degree or above. Paternal smoking history and pregnancy complications were coded as binary variables (yes/no) based on guardian reports.

### 2.4 Statistical analysis

Raw data were entered in Microsoft Excel and checked for logical inconsistencies and abnormal coding. Categorical variables were recoded according to the questionnaire and study definitions. Continuous variables included age, height, weight, BMI, locomotor skill score, ball skill score, and total FMS score. Participant characteristics were summarized descriptively. Continuous variables are presented as the median and interquartile range, M [P25, P75], and between-group comparisons were performed using the Mann–Whitney U-test. Categorical variables are presented as n (%), with between-group comparisons performed using the chi-squared test or Fisher’s exact test when expected cell counts did not satisfy the assumptions of the chi-squared test. A multivariable logistic regression analysis was performed to examine the association between insufficient infant crawling experience and relatively insufficient FMS development in preschool children. Three models were constructed with sequential adjustment for potential confounders: Model 1 adjusted for age, sex, and BMI; Model 2 additionally adjusted for gestational age, feeding mode, and mode of delivery; and Model 3 additionally adjusted for highest parental educational level, paternal smoking history, and pregnancy complications. Regression results are reported as odds ratios (ORs), 95% confidence intervals (CIs), and *p*-values.

### 2.5 Sensitivity analysis

Sensitivity analyses were performed to evaluate the robustness of the primary regression results. For continuous variables included in the models, outliers were identified using Tukey’s interquartile range rule, with values outside [Q1–1.5 × IQR, Q3 + 1.5 × IQR] considered outliers. ORs, 95% CIs, and *p*-values before and after the exclusion of outliers were compared to assess the stability of the primary findings. Similar directions and statistical significance in the sensitivity analyses were interpreted as suggesting that the main conclusions were not materially driven by outliers. To evaluate the discriminative ability of the logistic regression models for relatively insufficient FMS development, ROC curves were generated from predicted probabilities for Models 1–3, and the area under the curve (AUC) was calculated. AUC values closer to 1 indicate better model discrimination.

## 3 Results

### 3.1 Participant characteristics

A total of 557 children were included: 195 (35.0%) in the insufficient crawling experience group and 362 (65.0%) in the relatively sufficient crawling experience group. Among the 195 children with insufficient crawling experience, 51 had never crawled, and 144 had crawled but met at least one criterion for insufficient experience. The groups differed significantly in age, height, gestational age, and paternal smoking history (all *p* < 0.05). The median height was 113.5 cm [107.5, 119.0] in the insufficient crawling experience group and 117.4 cm [110.7, 121.9] in the relatively sufficient group (*p* < 0.001). The insufficient crawling experience group had lower locomotor skill, ball skill, and total FMS scores than the relatively sufficient group (26.00 vs. 32.00, 21.00 vs. 30.00, and 47.00 vs. 61.00, respectively, all *p* < 0.001) (Table 1). Baseline characteristics of the sensitivity-analysis sample were generally consistent with those of the primary analysis and are presented in Supplementary Table S1.

**Table 1:** Participant characteristics according to infant crawling experience.

Variable	Overall (*n* = 557)	Insufficient crawling experience (*n* = 195)	Relatively sufficient crawling experience (*n* = 362)	*p*
Age, years, median [P25, P75]	5.0 [4.0, 6.0]	5.0 [4.0, 5.0]	5.0 [4.0, 6.0]	<0.001
Sex (boys), *n* (%)	295 (53.0)	100 (51.3)	195 (53.9)	0.621
Height, cm, median [P25, P75]	115.5 [110.0, 121.0]	113.5 [107.5, 119.0]	117.4 [110.7, 121.9]	<0.001
BMI, kg/m^2^, median [P25, P75]	15.39 [14.38, 16.72]	15.39 [14.47, 16.71]	15.37 [14.36, 16.74]	0.863
Gestational age, *n* (%)				<0.001
<37 weeks	21 (3.8)	15 (7.7)	6 (1.7)	
37–41 weeks	488 (87.6)	160 (82.1)	328 (90.6)	
≥42 weeks	48 (8.6)	20 (10.3)	28 (7.7)	
Feeding mode, *n* (%)				0.147
Breastfeeding	323 (58.0)	116 (59.5)	207 (57.2)	
Formula feeding	46 (8.3)	21 (10.8)	25 (6.9)	
Mixed feeding	188 (33.8)	58 (29.7)	130 (35.9)	
Mode of delivery, *n* (%)				0.134
Vaginal delivery	246 (44.2)	95 (48.7)	151 (41.7)	
Cesarean delivery	311 (55.8)	100 (51.3)	211 (58.3)	
Highest parental education, *n* (%)				0.350
Junior high school or below	72 (12.9)	26 (13.3)	46 (12.7)	
High school/technical secondary school	190 (34.1)	57 (29.2)	133 (36.7)	
College or university	225 (40.4)	85 (43.6)	140 (38.7)	
Master’s degree or above	70 (12.6)	27 (13.8)	43 (11.9)	
Paternal smoking history (yes), *n* (%)	176 (31.6)	78 (40.0)	98 (27.1)	0.002
Pregnancy complications (yes), *n* (%)	120 (21.5)	42 (21.5)	78 (21.5)	1.000
Locomotor skill score, median [P25, P75]	30.00 [25.00, 34.00]	26.00 [21.00, 29.00]	32.00 [28.00, 35.00]	<0.001
Ball skill score, median [P25, P75]	26.00 [21.00, 32.00]	21.00 [17.00, 26.00]	30.00 [24.00, 35.00]	<0.001
Total FMS score, median [P25, P75]	56.00 [47.00, 65.00]	47.00 [40.00, 52.50]	61.00 [54.00, 69.00]	<0.001
Ever crawled, *n* (%)				<0.001
Yes	506 (90.8)	144 (73.8)	362 (100.0)	
No	51 (9.2)	51 (26.2)	0 (0.0)	
Daily crawling duration, *n* (%)				<0.001
No crawling	51 (9.2)	51 (26.2)	0 (0.0)	
<1 h/day	145 (26.0)	60 (30.8)	85 (23.5)	
1–2 h/day	201 (36.1)	59 (30.3)	142 (39.2)	
2–3 h/day	109 (19.6)	25 (12.8)	84 (23.2)	
≥3 h/day	51 (9.2)	0 (0.0)	51 (14.1)	
Crawling posture, *n* (%)				<0.001
No crawling	51 (9.2)	51 (26.2)	0 (0.0)	
Single posture	310 (55.7)	135 (69.2)	175 (48.3)	
Multiple postures	196 (35.2)	9 (4.6)	187 (51.7)	
Crawling motivation, *n* (%)				<0.001
No crawling	51 (9.2)	51 (26.2)	0 (0.0)	
Active crawling	334 (60.0)	96 (49.2)	238 (65.7)	
Passively guided crawling	13 (2.3)	5 (2.6)	8 (2.2)	
Both active and passive	159 (28.5)	43 (22.1)	116 (32.0)	
Crawling duration, *n* (%)				<0.001
No crawling	51 (9.2)	51 (26.2)	0 (0.0)	
<2 months	77 (13.8)	30 (15.4)	47 (13.0)	
2–3 months	122 (21.9)	35 (17.9)	87 (24.0)	
3–4 months	135 (24.2)	41 (21.0)	94 (26.0)	
4–5 months	116 (20.8)	32 (16.4)	84 (23.2)	
≥5 months	56 (10.1)	6 (3.1)	50 (13.8)	
Backward crawling, *n* (%)				<0.001
No crawling	51 (9.2)	51 (26.2)	0 (0.0)	
Yes	256 (46.0)	70 (35.9)	186 (51.4)	
No	250 (44.9)	74 (37.9)	176 (48.6)	
Crawling while carrying objects, *n* (%)				<0.001
No crawling	51 (9.2)	51 (26.2)	0 (0.0)	
Yes	229 (41.1)	66 (33.8)	163 (45.0)	
No	277 (49.7)	78 (40.0)	199 (55.0)	

Continuous variables are presented as the median [P25, P75] and compared using the Mann–Whitney U-test. Categorical variables are presented as *n* (%) and compared using the chi-squared test or Fisher’s exact test, as appropriate.

### 3.2 Multivariable logistic regression analysis

The multivariable logistic regression analysis showed that, after adjustment for age, sex, and BMI, insufficient infant crawling experience was significantly associated with relatively insufficient FMS development in preschool children. Compared with children with relatively sufficient crawling experience, children with insufficient crawling experience had higher odds of being classified as having relatively insufficient FMS (OR = 7.82, 95% CI: 4.54–13.48, *p* < 0.001). After additional adjustment for gestational age, feeding mode, and mode of delivery, the association remained statistically significant (OR = 7.81, 95% CI: 4.47–13.64, *p* < 0.001). The fully adjusted model yielded a similar result (OR = 8.36, 95% CI: 4.67–14.95, *p* < 0.001) (Table 2). In the sensitivity analysis excluding outliers, the direction and statistical significance of the association were consistent with the primary analysis across all three models, with ORs of 6.59 (95% CI: 3.74–11.61), 6.71 (95% CI: 3.77–11.94), and 7.34 (95% CI: 4.03–13.39), respectively (all *p* < 0.001), suggesting that the primary findings were not materially affected by outliers (Table 3).

**Table 2:** Multivariable logistic regression model results of insufficient infant crawling experience and relatively insufficient FMS development.

Model	β	SE	Wald χ^2^	OR (95% CI)	*p*
Model 1	2.057	0.278	54.908	7.82 (4.54, 13.48)	<0.001
Model 2	2.055	0.285	52.137	7.81 (4.47, 13.64)	<0.001
Model 3	2.123	0.297	51.261	8.36 (4.67, 14.95)	<0.001

Outcome coding: 0 = not relatively insufficient FMS development; 1 = relatively insufficient FMS development. Exposure coding: 0 = relatively sufficient crawling experience; 1 = insufficient crawling experience.

**Table 3:** Multivariable logistic regression model results after excluding outliers.

Model	β	SE	Wald χ^2^	OR (95% CI)	*p*
Model 1	1.886	0.289	42.584	6.59 (3.74, 11.61)	<0.001
Model 2	1.904	0.294	41.993	6.71 (3.77, 11.94)	<0.001
Model 3	1.994	0.306	42.334	7.34 (4.03, 13.39)	<0.001

Outcome coding: 0 = not relatively insufficient FMS development; 1 = relatively insufficient FMS development. Exposure coding: 0 = relatively sufficient crawling experience; 1 = insufficient crawling experience.

### 3.3 Exploratory analysis of model discrimination

To further evaluate the ability of the different logistic regression models to discriminate between children with and without relatively insufficient FMS development in preschool children, ROC curves were generated for both the primary-analysis sample and the sensitivity-analysis sample after the exclusion of outliers. In the primary analysis, the AUCs for Models 1, 2, and 3 were 0.884, 0.887, and 0.895, respectively. After the exclusion of outliers, the corresponding AUCs were 0.883, 0.888, and 0.893. Thus, the fitted models showed relatively high discrimination within the present sample, with similar AUC estimates before and after the exclusion of outliers. Because this was an exploratory analysis conducted in the same study sample and no internal or external validation was performed, these AUC estimates should not be interpreted as validated predictive performance (Figure 1).

**Figure 1:**
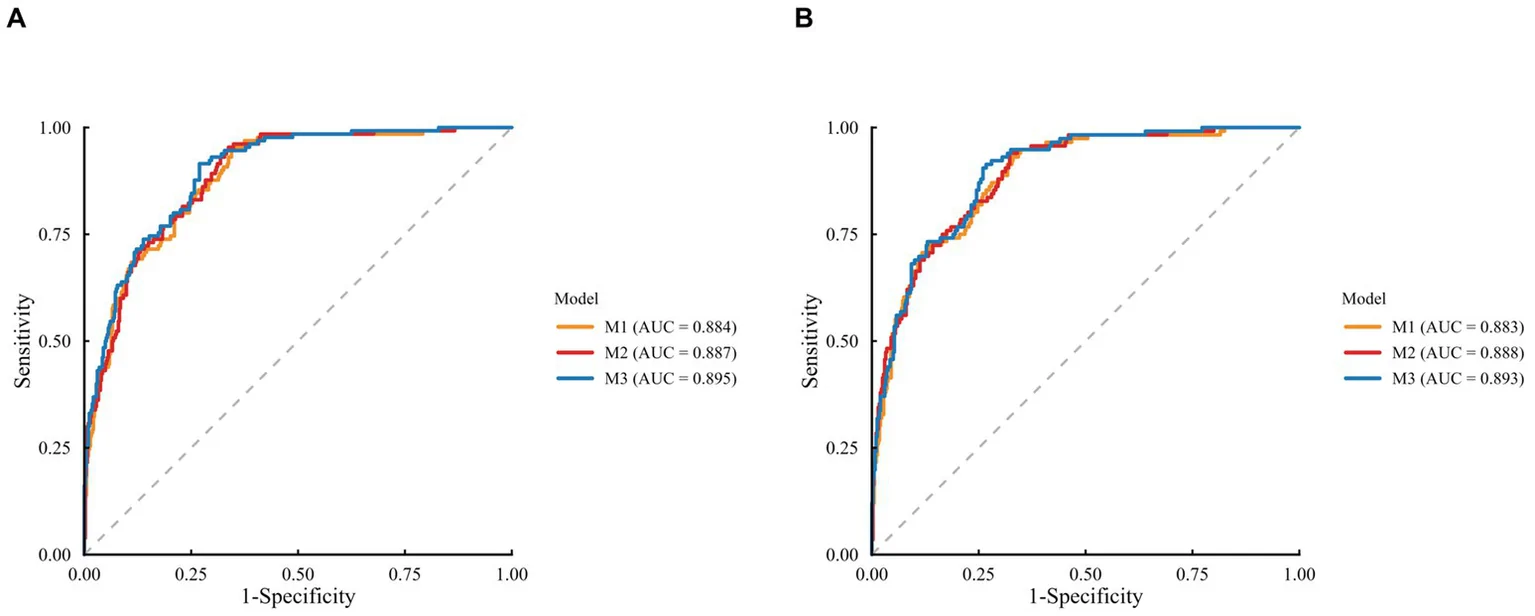
ROC curves for different logistic regression models. **(A)** ROC curves for the primary-analysis sample; **(B)** ROC curves for the sensitivity-analysis sample after the exclusion of outliers.

### 3.4 Supplementary analyses of crawling characteristics

Using daily crawling ≥3 h/day as the reference category, the 1–2 h/day, <1 h/day, and no-crawling groups had significantly higher odds of being classified as having relatively insufficient FMS development, with ORs of 5.98, 13.08, and 8.83, respectively; the trend test for daily crawling duration was statistically significant (*p* for trend < 0.001), whereas the 2–3 h/day group did not differ significantly from the reference group. Using crawling duration ≥5 months as the reference category, only the 2–3 month group had significantly higher odds (OR = 3.72, 95% CI: 1.26–10.94, *p* = 0.017). The overall trend test was significant (*p* for trend = 0.005), but the category-specific ORs did not show a fully monotonic dose–response gradient. Among children with crawling experience, multiple crawling postures were associated with lower odds of being classified as having relatively insufficient FMS development (OR = 0.11, 95% CI: 0.05–0.25, *p* < 0.001). The association for passively guided crawling was at the threshold of statistical significance (*p* = 0.050), whereas other crawling-motivation categories, backward crawling, and crawling while carrying objects were not significantly associated with the outcomes. The results for Models 1 and 2 are presented in Supplementary Tables S2 and S3.

## 4 Discussion

The present study found a significant association between insufficient infant crawling experience and relatively insufficient FMS development in preschool children. The adjusted association was consistent with our primary hypothesis and remained similar in direction and statistical significance in the sensitivity analysis. However, this consistency reflects a population-level statistical association and does not establish a causal developmental pathway. Previous research has shown that crawling and walking infants differ in their visual experiences during natural locomotion (Kretch et al., 2014), and independent walking experience is also closely associated with infants’ locomotor exploration (Briet et al., 2025), suggesting that early self-produced locomotor experience may be associated with how infants explore the environment and obtain information. Infant gross motor milestones have also been associated with subsequent development, later sports participation, and childhood physical activity (Ghassabian et al., 2016; Ridgway et al., 2009; Aoyama et al., 2023), broadly consistent with the direction of our findings. One possible interpretation is that crawling provides repeated opportunities for postural control, weight shifting, interlimb coordination, and visuospatial exploration, capacities that may also be relevant to later locomotor and ball skills. However, this interpretation remains theoretical because these intermediate sensorimotor processes were not directly measured in this study, and the available evidence does not establish that crawling experience itself directly influences later FMS. The observed association may also be influenced by underlying differences in early neuromotor development and by family- and caregiving-related factors. Previous studies have suggested that parent–child interaction, family type, and feeding mode are associated with early gross motor development (Xiong et al., 2024). Residual confounding therefore cannot be excluded, and the findings should not be interpreted directionally or causally.

Analyses of specific crawling characteristics showed that daily crawling duration was associated with relatively insufficient FMS development in a trend analysis. Although the overall trend test for the interval from crawling onset to independent walking was also statistically significant (p for trend = 0.005), the category-specific ORs did not change monotonically and therefore did not support a clear dose–response relationship. Among children with crawling experience, multiple crawling postures were associated with lower odds of being classified as having relatively insufficient FMS development. Similarly, previous research has indicated substantial individual variability in infant crawling patterns and has suggested that crawling patterns and variability may differ according to developmental status (Yamamoto et al., 2023). In contrast, crawling motivation, backward crawling, and crawling while carrying objects did not show stable associations. These findings indicate that a single behavioral characteristic may be insufficient to characterize infant self-produced locomotor experience. Research on early motor development may therefore benefit from considering not only the timing of milestone attainment but also the sufficiency and diversity of self-produced locomotor experience. Overall, the supplementary analyses yielded mixed findings rather than consistent evidence across all crawling characteristics (Table 4).

**Table 4:** Fully adjusted logistic regression model results of infant crawling characteristics and relatively insufficient FMS development.

Variable	Level	Relatively insufficient FMS (%)	OR (95% CI)	*p*
Daily crawling duration	≥3 h/day	3.9	1.00 (reference)	—
	2–3 h/day	16.5	4.108 (0.753–22.412)	0.103
	1–2 h/day	18.9	5.982 (1.151–31.105)	0.033
	<1 h/day	36.6	13.080 (2.517–67.984)	0.002
	No crawling	37.3	8.829 (1.532–50.873)	0.015
Crawling duration	≥5 months	10.7	1.00 (reference)	—
	4–5 months	12.9	1.088 (0.335–3.531)	0.889
	3–4 months	17.0	1.900 (0.623–5.790)	0.259
	2–3 months	36.1	3.716 (1.262–10.942)	0.017
	<2 months	29.9	2.615 (0.830–8.241)	0.101
	No crawling	37.3	2.737 (0.813–9.219)	0.104
Crawling posture	Single posture	33.2	1.00 (reference)	—
	Multiple postures	4.1	0.111 (0.048–0.254)	<0.001
Crawling motivation	Active crawling	21.3	1.00 (reference)	—
	Passively guided crawling	46.2	4.467 (0.997–20.011)	0.050
	Both active and passive	21.4	0.816 (0.463–1.437)	0.482
Backward crawling	No	20.4	1.00 (reference)	—
	Yes	23.4	1.338 (0.799–2.241)	0.268
Crawling while carrying objects	No	24.9	1.00 (reference)	—
	Yes	18.3	0.683 (0.403–1.160)	0.159

The dependent variable was relatively insufficient FMS development, coded as 1 for a total FMS score <47 and 0 for a total FMS score ≥47.

Interpretation of the findings is constrained by the study design and measurement approach. Infant crawling information was based on guardians’ retrospective reports several years later, and daily duration, posture, and other behavioral details may therefore be subject to recall bias and exposure misclassification. Although age and BMI were included in the adjusted models, height differed significantly between the two groups and was not included as a separate covariate. Residual confounding related to growth or maturation therefore cannot be excluded. Some behavioral subgroups were small, and potentially relevant factors such as floor surface, clothing restrictions, childrearing practices, opportunities for self-produced movement at home, and broader cultural context were not directly measured. These unmeasured environmental and cultural factors may influence both infant crawling experience and later opportunities for motor practice and may therefore confound the observed associations; accordingly, the adjusted estimates should not be interpreted as independent effects of crawling experience itself. The sample was drawn primarily from kindergartens in Qufu, Shandong Province, and the surrounding areas, which may limit generalizability. This cautious interpretation is consistent with recent evidence. Kretch et al. (2024) found that pediatric physical therapists generally consider crawling important for child development, while also emphasizing the need for stronger empirical evidence to support crawling-related professional beliefs and clinical practices. Furthermore, a systematic review and meta-analysis has shown that the pooled effect size for delayed crawling was smaller than that for delayed independent walking in neurodevelopmental conditions, suggesting that delayed crawling may be a comparatively weaker developmental marker (Bowler et al., 2024). Future research may use prospective longitudinal follow-up embedded within child health and developmental surveillance systems (Lipkin et al., 2020; Zubler et al., 2022) and combine wearable devices and intelligent sensor technologies to obtain continuous, objective recordings of infant posture, self-produced locomotion, and movement behavior in natural environments (Airaksinen et al., 2020; Jeong et al., 2021; Airaksinen et al., 2022; Deng et al., 2025). At present, the findings are best regarded as hypothesis-generating evidence and are insufficient to support the use of insufficient crawling experience, crawling duration, or crawling posture as stand-alone clinical screening or diagnostic indicators. Similarly, the exploratory ROC analysis reflects only apparent discrimination within the present sample and does not constitute validation of a clinical prediction or screening model. The clinical and public-health relevance of these findings requires further confirmation in prospective studies and independent populations.

## Data Availability

The original contributions presented in the study are included in the article/Supplementary material, further inquiries can be directed to the corresponding author.
