# Supplementary material for: Association between infant crawling characteristics and fundamental movement skill development in preschool children
Source: Front Psychol. 2026 Oct 15;17:1944438. doi: 10.3389/fpsyg.2026.1944438 (PMC13716658; doi:10.3389/fpsyg.2026.1944438)
Supplement: Supplementary file 1 [file Table_1.docx]

Supplementary Material

**Associations of Infant Crawling Characteristics with Fundamental Movement Skill Development in Preschool Children**

*Supplementary tables and analyses*

Supplementary Analysis 1. Sensitivity Analysis After Excluding Outliers

Supplementary Table S1. Characteristics of the study population after excluding outliers, according to infant crawling status

| **Variable** | **Overall (n = 516)** | **Insufficient crawling experience (n = 177)** | **Adequate crawling experience (n = 339)** | **P value** |
| --- | --- | --- | --- | --- |
| Age, years, median [P25, P75] | 5.00 [4.00, 6.00] | 5.00 [4.00, 5.00] | 5.00 [4.00, 6.00] | <0.001 |
| Sex (male), n (%) | 268 (51.9) | 91 (51.4) | 177 (52.2) | 0.863 |
| Height, cm, median [P25, P75] | 115.20 [110.00, 120.40] | 113.20 [107.50, 118.00] | 116.80 [110.50, 121.50] | <0.001 |
| BMI, kg/m², median [P25, P75] | 15.28 [14.33, 16.51] | 15.28 [14.41, 16.60] | 15.26 [14.33, 16.46] | 0.933 |
| **Gestational age, n (%)** |  |  |  | **0.045** |
| ≤36 weeks | 15 (2.9) | 9 (5.1) | 6 (1.8) |  |
| 37–41 weeks | 456 (88.4) | 149 (84.2) | 307 (90.6) |  |
| ≥42 weeks | 45 (8.7) | 19 (10.7) | 26 (7.7) |  |
| **Feeding pattern, n (%)** |  |  |  | **0.378** |
| Breastfeeding | 298 (57.8) | 106 (59.9) | 192 (56.6) |  |
| Formula feeding | 42 (8.1) | 17 (9.6) | 25 (7.4) |  |
| Mixed feeding | 176 (34.1) | 54 (30.5) | 122 (36.0) |  |
| **Mode of delivery, n (%)** |  |  |  | **0.144** |
| Vaginal delivery | 225 (43.6) | 85 (48.0) | 140 (41.3) |  |
| Cesarean delivery | 291 (56.4) | 92 (52.0) | 199 (58.7) |  |
| **Highest parental education, n (%)** |  |  |  | **0.209** |
| Junior high school or below | 70 (13.6) | 25 (14.1) | 45 (13.3) |  |
| High school or technical secondary school | 174 (33.7) | 49 (27.7) | 125 (36.9) |  |
| College or university | 207 (40.1) | 79 (44.6) | 128 (37.8) |  |
| Master's degree or above | 65 (12.6) | 24 (13.6) | 41 (12.1) |  |
| Paternal smoking (yes), n (%) | 159 (30.8) | 66 (37.3) | 93 (27.4) | 0.021 |
| Pregnancy complications (yes), n (%) | 112 (21.7) | 37 (20.9) | 75 (22.1) | 0.750 |
| Physical fitness score, median [P25, P75] | 73.75 [66.94, 78.50] | 64.75 [60.75, 70.75] | 76.75 [72.38, 80.00] | <0.001 |
| Locomotor skill score, median [P25, P75] | 30.00 [25.00, 34.00] | 26.00 [22.00, 29.00] | 31.00 [28.00, 35.00] | <0.001 |
| Object-control skill score, median [P25, P75] | 26.00 [21.00, 32.00] | 22.00 [17.00, 26.00] | 29.00 [24.00, 35.00] | <0.001 |
| Total FMS score, median [P25, P75] | 56.00 [47.00, 64.00] | 48.00 [41.00, 53.00] | 61.00 [53.50, 68.00] | <0.001 |
| **Ever crawled, n (%)** |  |  |  | **<0.001** |
| Yes | 470 (91.1) | 131 (74.0) | 339 (100.0) |  |
| No | 46 (8.9) | 46 (26.0) | 0 (0.0) |  |
| **Insufficient crawling experience, n (%)** |  |  |  | **—** |
| Yes | 177 (34.3) | 177 (100.0) | 0 (0.0) |  |
| No | 339 (65.7) | 0 (0.0) | 339 (100.0) |  |
| **Daily crawling duration, n (%)** |  |  |  | **<0.001** |
| Never crawled | 46 (8.9) | 46 (26.0) | 0 (0.0) |  |
| <1 h/day | 134 (26.0) | 54 (30.5) | 80 (23.6) |  |
| 1–2 h/day | 188 (36.4) | 55 (31.1) | 133 (39.2) |  |
| 2–3 h/day | 103 (20.0) | 22 (12.4) | 81 (23.9) |  |
| ≥3 h/day | 45 (8.7) | 0 (0.0) | 45 (13.3) |  |
| **Crawling posture, n (%)** |  |  |  | **<0.001** |
| Never crawled | 46 (8.9) | 46 (26.0) | 0 (0.0) |  |
| Single posture | 292 (56.6) | 123 (69.5) | 169 (49.9) |  |
| Multiple postures | 178 (34.5) | 8 (4.5) | 170 (50.1) |  |
| **Crawling motivation, n (%)** |  |  |  | **<0.001** |
| Never crawled | 46 (8.9) | 46 (26.0) | 0 (0.0) |  |
| Self-initiated crawling | 310 (60.1) | 87 (49.2) | 223 (65.8) |  |
| Caregiver-guided crawling | 10 (1.9) | 3 (1.7) | 7 (2.1) |  |
| Both self-initiated and caregiver-guided | 150 (29.1) | 41 (23.2) | 109 (32.2) |  |
| **Total crawling period, n (%)** |  |  |  | **<0.001** |
| Never crawled | 46 (8.9) | 46 (26.0) | 0 (0.0) |  |
| <2 months | 73 (14.1) | 28 (15.8) | 45 (13.3) |  |
| 2–3 months | 112 (21.7) | 32 (18.1) | 80 (23.6) |  |
| 3–4 months | 124 (24.0) | 38 (21.5) | 86 (25.4) |  |
| 4–5 months | 109 (21.1) | 27 (15.3) | 82 (24.2) |  |
| ≥5 months | 52 (10.1) | 6 (3.4) | 46 (13.6) |  |
| **Backward crawling, n (%)** |  |  |  | **<0.001** |
| Never crawled | 46 (8.9) | 46 (26.0) | 0 (0.0) |  |
| Yes | 235 (45.5) | 63 (35.6) | 172 (50.7) |  |
| No | 235 (45.5) | 68 (38.4) | 167 (49.3) |  |
| **Carrying objects while crawling, n (%)** |  |  |  | **<0.001** |
| Never crawled | 46 (8.9) | 46 (26.0) | 0 (0.0) |  |
| Yes | 218 (42.2) | 61 (34.5) | 157 (46.3) |  |
| No | 252 (48.8) | 70 (39.5) | 182 (53.7) |  |

**Note.** Values are presented as median [25th percentile, 75th percentile] or n (%), as appropriate. Continuous variables were compared using the Mann–Whitney U test; categorical variables were compared using the chi-square test or Fisher’s exact test. BMI, body mass index; FMS, fundamental movement skills.

After excluding outliers, 516 preschool children were included in the sensitivity analysis, including 177 children in the crawling-deficiency group and 339 children in the normal-crawling group. No statistically significant differences were observed between groups in sex distribution, BMI, feeding pattern, delivery mode, highest parental education, or pregnancy complications (all P > 0.05). Compared with children in the normal-crawling group, children with crawling deficiency had lower height, a different gestational-age distribution, and a higher proportion of paternal smoking (P < 0.05).

For motor-development indicators, the crawling-deficiency group had lower physical fitness scores, locomotor skill scores, object-control skill scores, and total FMS scores than the normal-crawling group (all P < 0.001). Significant between-group differences were also observed in crawling experience, daily crawling duration, total crawling period, crawling posture, crawling motivation, crawling regression, and carrying objects while crawling (all P < 0.001). Overall, children with crawling deficiency showed lower motor-development performance and less sufficient crawling-related experience.

Supplementary Table S2. Multivariable logistic regression analysis of infant crawling behavior characteristics and relative FMS underdevelopment: Model 1

| **Variable** | **Category** | **Relative underdevelopment (%)** | **OR (95% CI)** | **P value** |
| --- | --- | --- | --- | --- |
| **Daily crawling duration** | **≥3 h/day** | **3.9** | **1.00 (reference)** | **—** |
|  | 2–3 h/day | 16.5 | 4.652(0.916–23.632) | 0.064 |
|  | 1–2 h/day | 18.9 | 6.728(1.385–32.685) | 0.018 |
|  | ≤1 h/day | 36.6 | 15.130(3.118–73.427) | 0.001 |
|  | Never crawled | 37.3 | 10.347(1.948–54.971) | 0.006 |
|  | P for trend |  |  | <0.001 |
| **Total crawling period** | **≥5 months** | **10.7** | **1.00 (reference)** | **—** |
|  | 4–5 months | 12.9 | 1.537(0.498–4.740) | 0.454 |
|  | 3–4 months | 17.0 | 2.185(0.745–6.406) | 0.154 |
|  | 2–3 months | 36.1 | 4.321(1.515–12.326) | 0.006 |
|  | <2 months | 29.9 | 3.363(1.110–10.184) | 0.032 |
|  | Never crawled | 37.3 | 3.485(1.095–11.097) | 0.035 |
|  | P for trend |  |  | 0.002 |
| **Crawling posture** | **Single posture** | **33.2** | **1.00 (reference)** | **—** |
|  | Multiple postures | 4.1 | 0.113(0.051–0.250) | <0.001 |
| **Crawling motivation** | **Self-initiated crawling** | **21.3** | **1.00 (reference)** | **—** |
|  | Caregiver-guided crawling | 46.2 | 4.270(1.008–18.091) | 0.049 |
|  | Both self-initiated and caregiver-guided | 21.4 | 0.778(0.453–1.337) | 0.364 |
| **Backward crawling** | **No** | **20.4** | **1.00 (reference)** | **—** |
|  | Yes | 23.4 | 1.265(0.772–2.073) | 0.351 |
| **Carrying objects while crawling** | **No** | **24.9** | **1.00 (reference)** | **—** |
|  | Yes | 18.3 | 0.649(0.393–1.072) | 0.091 |

**Note.** The outcome was relative FMS underdevelopment (total FMS score <47: coded 1; score ≥47: coded 0). Each crawling characteristic was entered into a separate multivariable logistic regression model. Model 1 adjusted for age, sex, and body mass index. Daily crawling duration and total crawling period were analyzed in the full sample; crawling posture, crawling motivation, backward crawling, and carrying objects while crawling were analyzed only among children with a crawling experience. Trend tests treated the ordered categories as continuous scores. OR, odds ratio; CI, confidence interval.

Supplementary Table S3. Multivariable logistic regression analysis of infant crawling behavior characteristics and relative FMS underdevelopment: Model 2

| **Variable** | **Category** | **Relative underdevelopment (%)** | **OR (95% CI)** | **P value** |
| --- | --- | --- | --- | --- |
| **Daily crawling duration** | **≥3 h/day** | **3.9** | **1.00 (reference)** | **—** |
|  | 2–3 h/day | 16.5 | 4.859(0.893–26.422) | 0.067 |
|  | 1–2 h/day | 18.9 | 6.943(1.334–36.142) | 0.021 |
|  | ≤1 h/day | 36.6 | 15.771(3.041–81.784) | 0.001 |
|  | Never crawled | 37.3 | 10.515(1.834–60.297) | 0.008 |
|  | P for trend |  |  | <0.001 |
| **Total crawling period** | **≥5 months** | **10.7** | **1.00 (reference)** | **—** |
|  | 4–5 months | 12.9 | 1.272(0.400–4.052) | 0.683 |
|  | 3–4 months | 17.0 | 2.241(0.755–6.649) | 0.146 |
|  | 2–3 months | 36.1 | 4.017(1.391–11.598) | 0.010 |
|  | <2 months | 29.9 | 3.254(1.049–9.866) | 0.041 |
|  | Never crawled | 37.3 | 3.141(0.956–10.321) | 0.059 |
|  | P for trend |  |  | 0.003 |
| **Crawling posture** | **Single posture** | **33.2** | **1.00 (reference)** | **—** |
|  | Multiple postures | 4.1 | 0.113(0.050–0.250) | <0.001 |
| **Crawling motivation** | **Self-initiated crawling** | **21.3** | **1.00 (reference)** | **—** |
|  | Caregiver-guided crawling | 46.2 | 4.270(0.985–18.475) | 0.052 |
|  | Both self-initiated and caregiver-guided | 21.4 | 0.814(0.466–1.422) | 0.470 |
| **Backward crawling** | **No** | **20.4** | **1.00 (reference)** | **—** |
|  | Yes | 23.4 | 1.268(0.767–2.095) | 0.355 |
| **Carrying objects while crawling** | **No** | **24.9** | **1.00 (reference)** | **—** |
|  | Yes | 18.3 | 0.672(0.400–1.128) | 0.132 |

**Note.** The outcome was relative FMS underdevelopment (total FMS score <47: coded 1; score ≥47: coded 0). Each crawling characteristic was entered into a separate multivariable logistic regression model. Model 2 adjusted for age, sex, body mass index, gestational age, feeding pattern, and mode of delivery. Daily crawling duration and total crawling period were analyzed in the full sample; crawling posture, crawling motivation, backward crawling, and carrying objects while crawling were analyzed only among children with a crawling experience. Trend tests treated the ordered categories as continuous scores. OR, odds ratio; CI, confidence interval.

In Model 1, after adjustment for age, sex, and body mass index, daily crawling duration was significantly associated with relative FMS underdevelopment. Compared with children who crawled for ≥3 h/day, those who crawled for 1–2 h/day, ≤1 h/day, or never crawled had significantly higher odds of relative FMS underdevelopment (ORs = 6.73, 15.13, and 10.35, respectively), with a significant trend across categories (P for trend <0.001). For total crawling period, children who crawled for 2–3 months, <2 months, or never crawled had significantly higher odds than those who crawled for ≥5 months (ORs = 4.32, 3.36, and 3.49, respectively; P for trend = 0.002). Among children with a crawling experience, multiple crawling postures were associated with substantially lower odds of relative FMS underdevelopment (OR = 0.11, 95% CI: 0.05–0.25; P <0.001). Caregiver-guided crawling showed a borderline association with higher odds, whereas the other crawling motivation category, backward crawling, and carrying objects while crawling were not significantly associated with the outcome.

After additional adjustment for gestational age, feeding pattern, and mode of delivery in Model 2, the overall findings remained stable. Crawling for 1–2 h/day, ≤1 h/day, or never crawling remained associated with higher odds of relative FMS underdevelopment (ORs = 6.94, 15.77, and 10.52, respectively; P for trend <0.001). Total crawling periods of 2–3 months and <2 months were also associated with higher odds (ORs = 4.02 and 3.25, respectively), whereas the association for never crawling was borderline (P = 0.059); the overall trend remained significant (P for trend = 0.003). The inverse association for multiple crawling postures remained significant, whereas crawling motivation, backward crawling, and carrying objects while crawling did not show stable significant associations.

Supplementary Table S4. Multivariable logistic regression analysis of absence of infant crawling experience and relative FMS underdevelopment

| **Model** | **β** | **SE** | **Wald χ²** | **OR** | **95% CI** | **P value** |
| --- | --- | --- | --- | --- | --- | --- |
| Model 1 | 0.313 | 0.369 | 0.722 | 1.37 | 0.66–2.82 | 0.396 |
| Model 2 | 0.262 | 0.386 | 0.460 | 1.30 | 0.61–2.77 | 0.498 |
| Model 3 | 0.244 | 0.393 | 0.383 | 1.28 | 0.59–2.76 | 0.536 |

**Note.** The outcome was relative FMS underdevelopment (total FMS score <47: coded 1; score ≥47: coded 0). The exposure was absence of crawling experience (ever crawled: coded 0; never crawled: coded 1), with children who had crawled as the reference. Model 1 adjusted for age, sex, and body mass index. Model 2 additionally adjusted for gestational age, feeding pattern, and mode of delivery. Model 3 additionally adjusted for highest parental education, paternal smoking, and pregnancy complications. β, regression coefficient; CI, confidence interval; OR, odds ratio; SE, standard error.

After adjustment for age, sex, and body mass index, children who had never crawled had higher odds of relative FMS underdevelopment than children with a crawling experience, although the association was not statistically significant (OR = 1.37, 95% CI: 0.66–2.82; P = 0.396). The association remained non-significant after additional adjustment for gestational age, feeding pattern, and mode of delivery (OR = 1.30, 95% CI: 0.61–2.77; P = 0.498), and after full adjustment for highest parental education, paternal smoking, and pregnancy complications (OR = 1.28, 95% CI: 0.59–2.76; P = 0.536).
